# Latent classes and symptom network analysis of kinesiophobia in older adult patients with chronic obstructive pulmonary disease

**DOI:** 10.3389/fmed.2026.1919256

**Published:** 2026-10-05

**Authors:** Mengyao Liang, Congying Kang, Li Feng, Jiajia Hua

**Affiliations:** 1 Department of Nursing, The Sixth People's Hospital of Nantong, Nantong, Jiangsu, China; 2 Department of Pulmonary Oncology, The Sixth People's Hospital of Nantong, Nantong, Jiangsu, China; 3 Department of Traditional Chinese Medicine, The Sixth People's Hospital of Nantong, Nantong, Jiangsu, China

**Keywords:** chronic obstructive pulmonary disease, kinesiophobia, latent profile analysis, older adults, pulmonary rehabilitation, symptom network analysis

## Abstract

**Objective:**

To identify the latent classes of kinesiophobia in older adult patients with chronic obstructive pulmonary disease (COPD), analyze the symptom network structure of different classes, determine the core symptoms, and provide evidence for precision pulmonary rehabilitation interventions.

**Methods:**

A convenience sample of 546 older adult COPD patients was recruited from the Department of Respiratory and Critical Care Medicine, Nantong Sixth People's Hospital, between March 2025 and April 2026. Participants completed a general information questionnaire, the Breathlessness Beliefs Questionnaire (BBQ), and a COPD symptom survey. Latent profile analysis was used to identify potential classes of kinesiophobia. Symptom networks for each subgroup were constructed using R software, and three centrality indices (strength, closeness, and betweenness) were calculated to determine core symptoms.

**Results:**

The prevalence of kinesiophobia in older adult patients with COPD was 88.46%. Latent profile analysis identified two classes: a high-fear-somatic focus group (67.4%) and a lower-fear-activity avoidance group (32.6%). Symptom network analysis revealed that the core symptom was dyspnea (strength = 1.78) in the high-fear group, whereas fatigue (strength = 1.64) was the core symptom in the lower-fear group. The difference in network structure between the two groups was not statistically significant (*P* > 0.05).

**Conclusion:**

Kinesiophobia in older adult patients with COPD exhibits population heterogeneity, with different core symptoms across subgroups. The findings of this study suggest that dyspnea deserves particular attention in the high-fear group, while fatigue deserves particular attention in the lower-fear group. Future longitudinal or interventional studies are needed to further verify the causal relationships between core symptoms and pulmonary rehabilitation participation, thereby informing the development of individualized pulmonary rehabilitation strategies.

## 1 Introduction

Chronic obstructive pulmonary disease (COPD) is one of the leading causes of morbidity and mortality worldwide, with a prevalence exceeding 13% among adults aged 40 years and older in China (1). Pulmonary rehabilitation (PR), particularly comprehensive interventions centered on exercise training, is the cornerstone of non-pharmacological management for COPD and has been proven to improve exercise tolerance, alleviate dyspnea symptoms, and reduce the frequency of acute exacerbations (2, 3). However, actual participation and adherence to PR remain far below optimal levels, with dropout rates reaching as high as 30%–50% in real-world settings (4, 5). Among the numerous barriers, kinesiophobia—defined as an excessive, irrational fear of physical activity due to anticipated dyspnea or discomfort—is increasingly recognized as a critical psychological obstacle (6). Unlike generalized anxiety, kinesiophobia in COPD patients is specifically linked to the expectation that “activity will trigger unbearable dyspnea,” leading to activity avoidance and further physical deconditioning, thereby forming a self-reinforcing vicious cycle (7). The fear-avoidance model, originally developed in the context of chronic pain, provides a coherent theoretical framework for understanding kinesiophobia in COPD (8). Dyspnea and pain partially share neural pathways and cortical projection areas. When patients catastrophically interpret dyspnea during physical activity, this may theoretically lead to maladaptive avoidance behaviors and further undermine their exercise self-efficacy. However, this pathway is difficult to directly examine in cross-sectional data. Recent evidence indicates that kinesiophobia is highly prevalent in older adult patients with COPD, but reported prevalence rates vary considerably depending on the assessment tool and cutoff criteria used. For instance, studies using the TSK with a high-level cutoff reported a prevalence of approximately 59.9%, while those using the BBQ with a total score >33 as the threshold reported prevalence rates as high as 88% (9, 10). Nevertheless, most studies to date have treated kinesiophobia as a homogeneous construct, simply using total scale score cutoffs to dichotomize patients into “high-fear” and “low-fear” groups. This approach obscures within-group heterogeneity: some patients exhibit pronounced avoidance behaviors despite relatively low fear levels (acknowledging the importance of exercise but avoiding it out of safety-seeking), whereas others display high levels of fear primarily driven by somatic symptoms such as dyspnea and fatigue (10). Latent profile analysis can empirically identify these subgroups without requiring predetermined cutoffs, while symptom network analysis offers a complementary perspective by mapping how individual symptoms interconnect and identifying the core driving symptoms within each subgroup (11).

In the field of COPD, several studies have used network analysis to depict symptom association patterns in patients, consistently finding that dyspnea, fatigue, and anxiety often show high centrality in symptom networks and may serve as key nodes maintaining the symptom network (12). In other respiratory disease populations, such as asthma, network analysis has also been applied to identify core symptoms and bridge relationships among symptoms (13). However, existing network studies in COPD have mostly analyzed patients as a single overall sample or stratified only by disease severity, and have not yet combined network analysis with kinesiophobia subtypes. Consequently, the clinical question of “what are the core symptoms specific to each kinesiophobia subtype” remains unanswered.

To date, no study has combined latent profile analysis with symptom network analysis to systematically characterize subtype features of kinesiophobia in older adult patients with COPD. Therefore, the present study aimed to: 1) identify latent subtypes of kinesiophobia in older adult patients with COPD using latent profile analysis; 2) construct symptom networks for each subtype and determine core symptoms via centrality indices; and 3) compare network structure and global connectivity strength between subtypes. It should be noted that the cross-sectional design can only reveal associations among symptoms and cannot establish causality. Accordingly, the main value of this study lies in providing preliminary clues for identifying core symptoms that may influence kinesiophobia and pulmonary rehabilitation participation, and in offering a hypothesis base for future longitudinal and interventional studies, rather than directly determining intervention targets. The findings are expected to inform the development of individualized intervention strategies targeting subtype-specific core symptoms in the future, thereby laying a foundation for reducing kinesiophobia levels and improving pulmonary rehabilitation participation and adherence.

## 2 Participants and methods

### 2.1 Participants

Using a convenience sampling method, older adult patients with COPD who were hospitalized or attended outpatient follow-up at the Department of Respiratory and Critical Care Medicine, Nantong Sixth People's Hospital, between March 2025 and April 2026 were recruited as survey participants.1) Inclusion criteria:① Age ≥ 60 years;② Diagnosed with COPD according to the Global Initiative for Chronic Obstructive Lung Disease (GOLD 2025) criteria and currently in a stable phase (14);③ Alert and oriented, able to complete the questionnaires independently or with minimal assistance from the researcher;④ Provided informed consent and voluntarily participated in this study.2) Exclusion criteria:①severe cardiovascular or cerebrovascular diseases; ②acute exacerbation phase (requiring intravenous corticosteroids or antibiotics within 48 h after admission); ③severe musculoskeletal or neurological diseases that preclude any form of physical activity;④malignant tumors or other end-stage diseases; ⑤cognitive impairment or diagnosed psychiatric disorders; and ⑥being bedridden or completely dependent on others for care. This study involved two primary analytical approaches: latent profile analysis (LPA) and symptom network analysis. According to Monte Carlo simulation recommendations (15), the required sample size for LPA is 10–20 times the number of observed variables. For symptom network analysis, at least 3–5 cases per estimated parameter are recommended. In this study, the symptom network comprised 15 nodes, yielding 15 × 14/2 = 105 partial correlation parameters to be estimated. At 5 cases per parameter, a minimum of 525 cases was required. After accounting for a 10% invalid response rate, the final target sample size was set at 580. A total of 546 valid questionnaires were ultimately collected, yielding an effective response rate of 94.1%. This study was approved by the Ethics Committee of Nantong Sixth People's Hospital (Approval No.: NTLYLL2025033). All patients provided signed informed consent.

### 2.2 Measurement instruments

#### 2.2.1 General information questionnaire

A self-designed general information questionnaire was used to collect sociodemographic characteristics (age, sex, marital status, educational level, and living situation) and disease-related characteristics (COPD duration, pulmonary function grade, number of acute exacerbations in the past year, smoking history, number of comorbidities, and whether the patient had received pulmonary rehabilitation guidance).

#### 2.2.2 Kinesiophobia assessment tool

The Breathlessness Beliefs Questionnaire (BBQ) was used to assess kinesiophobia in COPD patients. Developed by De Peuter et al. (16), the BBQ consists of 11 items divided into two dimensions: somatic focus (6 items) and activity avoidance (5 items). Each item is rated on a 5-point Likert scale (1 = strongly disagree, 5 = strongly agree), with total scores ranging from 11 to 55, where higher scores indicate greater kinesiophobia. The Chinese version of the BBQ has demonstrated good reliability and validity, with a Cronbach's *α* coefficient of 0.82 and test–retest intraclass correlation coefficients ranging from 0.96 to 0.98. In the pilot test of this study (*n* = 40), the Cronbach's *α* was 0.85.

#### 2.2.3 COPD symptom survey questionnaire

Based on a literature review and expert consultation, core items from the COPD Assessment Test (CAT) (17), the Modified Medical Research Council Dyspnoea Scale (mMRC) (18), the Hospital Anxiety and Depression Scale (HADS) (19), and the Fatigue Severity Scale (FSS) (18) were integrated to identify the 15 most common symptoms in COPD patients: dyspnea, cough, sputum production, chest tightness, fatigue, leg weakness, anxiety, depression, sleep disturbance, appetite loss, dry mouth, constipation, social avoidance, poor concentration, and palpitations. Each symptom was rated on a 5-point Likert scale (0 = none at all, 4 = very severe) to assess symptom severity over the past week. The questionnaire was evaluated for content validity by five experts in respiratory and critical care medicine and nursing. The item-level content validity index (I-CVI) ranged from 0.80 to 1.00, and the scale-level content validity index (S-CVI/Ave) was 0.92. In the pilot test (*n* = 40), the Cronbach's *α* was 0.84.

### 2.3 Data collection procedure

The questionnaire survey was conducted by two uniformly trained supervising nurses. The training covered the study objectives, mastery of the inclusion and exclusion criteria, standardized interpretation of each questionnaire item, instructions, and the survey procedure. A consistency assessment was conducted after the training, and only those who passed the assessment were allowed to participate in the survey. The survey was administered when hospitalized patients were clinically stable before discharge or during outpatient follow-up visits. It was conducted during relatively quiet periods in the hospital (e.g., 9:00–11:00 or 14:00–16:00), and all questionnaires were completed by the same investigator using a one-on-one, face-to-face approach. The two investigators randomly undertook the survey tasks, and each patient's questionnaires were completed by the same investigator throughout. Before the survey, the study purpose, significance, and confidentiality principles were explained to the patients, and questionnaires were distributed after written informed consent was obtained. For participants who were unable to complete the questionnaires independently due to visual impairment or limited literacy, the investigator read each item aloud in a neutral tone and completed the questionnaire on their behalf. All questionnaires were collected on site and checked for completeness; those with more than 20% missing items were excluded.

To assess the potential impact of hospitalization status on the study results, we compared differences between hospitalized and outpatient participants in age, sex, disease duration, lung function grade, mMRC score, and BBQ total score. The results showed no statistically significant differences between the two groups (*P* > 0.05).

### 2.4 Statistical analysis

SPSS 26.0 was used for data entry and descriptive statistical analysis. Continuous variables with a normal distribution were presented as mea*n* ± standard deviation and compared between groups using the independent samples t-test; continuous variables with a non-normal distribution were presented as median (interquartile range) and compared using the Mann–Whitney U test. Categorical variables were presented as frequencies and percentages and compared using the chi-square test or Fisher's exact test.Mplus 8.3 was used to construct latent profile models. The 11 BBQ item scores were used as observed indicators, and models with 1 to 4 classes were fitted sequentially. Model fit indices included the Akaike Information Criterion (AIC), Bayesian Information Criterion (BIC), and sample-size adjusted BIC (aBIC), with smaller values indicating better fit; an Entropy value closer to 1 indicated higher classification accuracy. The Lo–Mendell–Rubin likelihood ratio test (LMRT) and bootstrap likelihood ratio test (BLRT) were used to compare the k-class model with the (k-1)-class model, with *P* < 0.05 indicating that the k-class model was superior. The final number of classes was determined by combining theoretical interpretability and the practical meaning of each class. Class membership was assigned using the maximum posterior probability method, with each patient allocated to the class with the highest posterior probability. For network analysis, R 4.3.1 software was used with the qgraph package (version 1.9.8) and bootnet package (version 1.5.1) to construct Gaussian graphical models (GGMs) based on partial correlation matrices. The network nodes represented the 15 symptoms, and edges represented partial correlation coefficients between two symptoms (i.e., conditional correlations after controlling for all other symptoms). Blue solid lines indicated positive correlations, red dashed lines indicated negative correlations, and thicker edges indicated stronger correlations. The EBIC-glasso algorithm was used for network estimation, which regularizes the partial correlation matrix via the graphical LASSO and selects the optimal network model using the Extended Bayesian Information Criterion (EBIC), with the hyperparameter *γ* set to 0.5. Nonparametric bootstrap (1000 iterations) was used to assess the accuracy of edge weights, and case-dropping bootstrap was used to evaluate the stability of centrality indices. Three centrality indices were calculated for each node: strength, closeness, and betweenness. Because closeness and betweenness are generally less stable than strength, strength was used as the primary criterion for identifying core symptoms given its acceptable stability, while closeness and betweenness were reported for descriptive purposes only. A correlation stability coefficient (CS-coefficient) greater than 0.50 was considered good, and greater than 0.25 was considered acceptable. The NetworkComparisonTest (NCT) package was used to compare network structure (network invariance) and global strength invariance between subgroups, with 1000 permutations, and *P* < 0.05 was considered statistically significant. A fixed random seed [set.seed(20250416)] was set for all network analyses and permutation tests to ensure reproducibility.

## 3 Results

### 3.1 General characteristics of older adults with COPD

The 546 older adult COPD patients had a mean age of 71.34 ± 7.62 years (range: 60–89 years), a mean COPD duration of 8.67 ± 5.41 years (range: 1–35 years), and a mean body mass index (BMI) of 21.87 ± 3.26 kg/m^2^ (range: 16.2–28.7 kg/m^2^). Among them, 392 (71.8%) were male and 154 (28.2%) were female; 100 patients (18.3%) were aged ≥80 years. Detailed characteristics are presented in Table 1.

**Table 1:** General characteristics of older adults with COPD (*n* = 546).

Variable	Category	*n*	%
Sex	Male	392	71.8
	Female	154	28.2
Age (years, mean ± SD)	—	71.34 ± 7.62	—
Age group (years)	60–69	218	39.9
	70–79	228	41.8
	≥80	100	18.3
BMI (kg/m^2^, mean ± SD)	—	21.87 ± 3.26	—
Marital status	Married	448	82.1
	Widowed	86	15.8
	Divorced	12	2.2
Educational level	Primary school or below	165	30.2
	Junior high school	198	36.3
	Senior high school/vocational school	124	22.7
	College degree or above	59	10.8
Residence	Urban	237	43.4
	Rural	309	56.6
COPD duration (years, mean ± SD)	—	8.67 ± 5.41	—
Duration group (years)	<5	178	32.6
	5–10	224	41.0
	>10	144	26.4
GOLD pulmonary function grade ①	Grade I (FEV₁/FVC <0.70, FEV₁ ≥ 80% predicted)	68	12.5
	Grade II (50% ≤ FEV₁ < 80%)	203	37.2
	Grade III (30% ≤ FEV₁ < 50%)	198	36.3
	Grade IV (FEV₁ < 30%)	77	14.1
mMRC dyspnea grade ②	Grade 0–1	189	34.6
	Grade 2	217	39.7
	Grade ≥3	140	25.6
Acute exacerbations in past year	0	112	20.5
	1–2	276	50.5
	≥3	158	28.9
Smoking history ③	Never smoked	103	18.9
	Former smoker	187	34.2
	Current smoker	256	46.9
Major comorbidities ④	Hypertension	189	34.6
	Diabetes mellitus	102	18.7
	Coronary heart disease	87	15.9
	History of stroke	42	7.7
Received pulmonary rehabilitation guidance	Yes	134	24.5
	No	412	75.5
Monthly household income per capita (CNY)	<3,000	238	43.6
	3,000–5,000	196	35.9
	>5,000	112	20.5

① GOLD grades are based on the airflow limitation severity classification criteria of GOLD 2025. ② mMRC grades: Grade 0 = dyspnea only during strenuous exercise; Grade 1 = shortness of breath when walking fast or walking up a slight hill; Grade 2 = walks slower than peers of the same age due to shortness of breath or needs to stop for breath; Grade 3 = stops for breath after walking approximately 100 meters or after a few minutes on level ground; Grade 4 = too breathless to leave the house or becomes breathless when dressing or undressing. ③ “Former smoker” refers to having completely stopped smoking for ≥6 months after being diagnosed with COPD.④ Comorbidities were multiple-choice; the number of cases for each condition was counted independently, and the sum exceeds 546.

### 3.2 Kinesiophobia status in older adults with COPD

Among the 546 patients, the total BBQ score ranged from 23 to 51 (38.42 ± 6.79). Using a cutoff of >33 on the BBQ to indicate kinesiophobia (19), 483 patients (88.46%) were classified as having kinesiophobia. The two dimension scores were as follows: the somatic focus dimension score ranged from 14 to 30 (21.63 ± 4.12), with a mean item score of 3.61; the activity avoidance dimension score ranged from 9 to 25 (16.79 ± 3.81), with a mean item score of 3.36.

### 3.3 Latent profile analysis of kinesiophobia in older adults with COPD

Using the 11 item scores of the Breathlessness Beliefs Questionnaire as manifest indicators, latent profile models with one to four classes were sequentially fitted using Mplus 8.3. The model fit indices are presented in Table 2. As the number of classes increased, AIC, BIC, and aBIC continued to decrease. The two-class model had an entropy value of 0.943 (>0.90), indicating good classification accuracy; both LMRT and BLRT were *P* < 0.001, demonstrating that the two-class model was significantly superior to the one-class model. Although the three-class model had a slightly higher entropy value (0.958), both LMRT and BLRT were *P* > 0.05, and one class accounted for only 8.6% (47 cases), rendering it less clinically interpretable. The four-class model also failed the LMRT and BLRT tests. Considering both statistical indices and clinical meaningfulness, the two-class model was selected as the optimal model.

**Table 2:** Fit indices of latent profile models for kinesiophobia in older adults with COPD (*n* = 546).

Model	AIC	BIC	aBIC	Entropy	LMRT P	BLRT P	Class probabilities
1-class	18,234.56	18,401.23	18,278.91	—	—	—	—
**2-class**	**16,987**.**34**	**17,215**.**67**	**17,043**.**09**	**0**.**943**	**<0**.**001**	**<0**.**001**	**0.674/0.326**
3-class	16,542.10	16,831.59	16,609.78	0.958	0.087	0.089	0.521/0.393/0.086
4-class	16,331.22	16,681.03	16,410.07	0.965	0.142	0.138	0.408/0.305/0.179/0.108

Note.​ AIC = Akaike Information Criterion; BIC = Bayesian Information Criterion; aBIC = sample-size-adjusted Bayesian Information Criterion; Entropy = classification precision index (range 0-1, with values >0.8 indicating acceptable classification accuracy); LMRT = Lo-Mendell-Rubin likelihood ratio test; BLRT = bootstrap likelihood ratio test; Class probabilities = proportion of participants assigned to each latent class. Lower AIC, BIC, and aBIC values indicate better model fit. A significant LMRT or BLRT (p < .05) indicates that the k-class model fits significantly better than the (k−1)-class model.

The response patterns of the two latent classes on the 11 BBQ items are illustrated in Figure 1. Based on the distribution characteristics of item scores, the two classes were named as follows:

**Figure 1:**
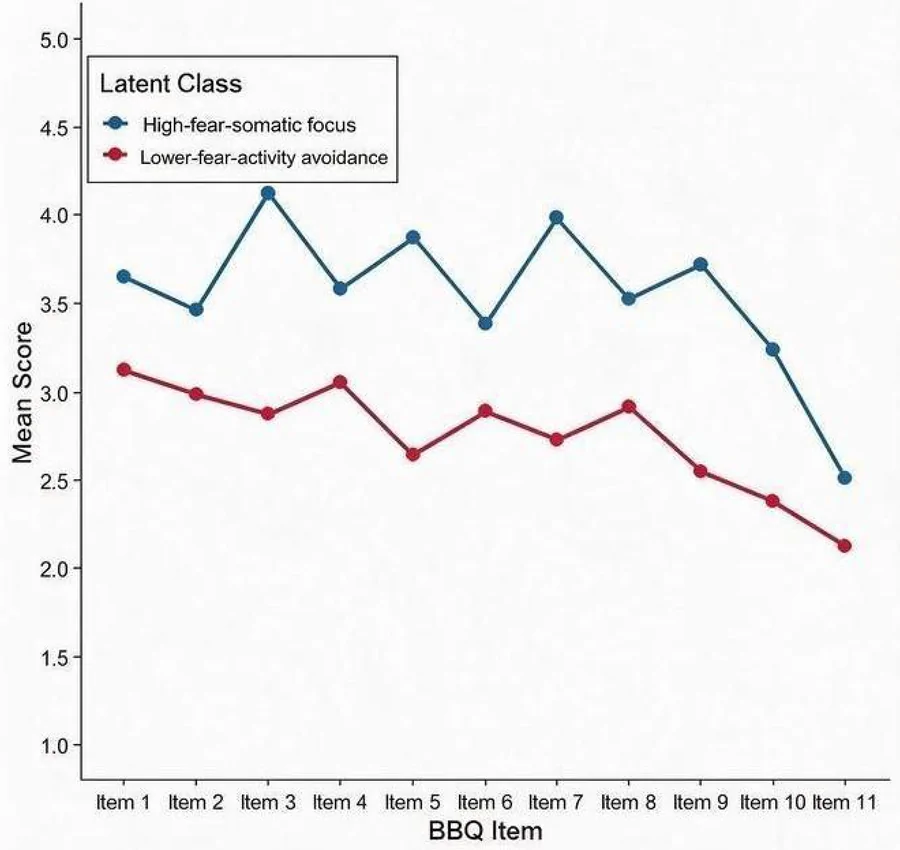
Two latent classes of kinesiophobia in older adults with COPD. S1 — Dyspnea; S2 — Fatigue; S3 — Cough; S4 — Sputum; S5 — Chest tightness; S6 — Leg weakness; S7 — Anxiety; S8 — Sleep disturbance; S9 — Appetite loss; S10 — Dry mouth; S11 — Social avoidance; S12 — Depression; S13 — Poor concentration; S14 — Constipation; S15 — Palpitations.

Class 1 (high-fear–somatic focus group): 368 patients (67.4%), with a mean BBQ total score of 43.87 ± 3.12. This group scored higher on item 3 (“When I feel short of breath during activity, I worry that my lungs are being damaged”), item 5 (“Exercise makes me feel more breathless”), item 7 (“Feeling short of breath during activity means my condition is worsening”), item 9 (“Exercise will aggravate my illness”), and item 11 (“Once I feel short of breath, I should stop activity immediately”). These items belong to the somatic focus dimension, which measures catastrophic cognition about dyspnea, suggesting that patients in this class tended to interpret exertional dyspnea as a dangerous signal of physical injury or disease progression, thereby developing strong kinesiophobia.

Class 2 (lower-fear–activity avoidance group): 178 patients (32.6%), with a mean BBQ total score of 35.16 ± 2.89. Although the total BBQ score in this group was still above the threshold for kinesiophobia (>33), their fear level was significantly lower than that of Class 1; therefore, this class was named the “lower-fear–activity avoidance group.”

Class membership was assigned using the maximum posterior probability method. Specifically, each patient was allocated to the class with the highest posterior probability estimated by the latent profile model. The average posterior probabilities for the two classes were 0.96 (high-fear–somatic focus group) and 0.93 (lower-fear–activity avoidance group), with an overall average posterior probability of 0.95, indicating high classification accuracy and low classification uncertainty. The high average posterior probabilities and entropy value (0.943) suggest that classification uncertainty is unlikely to have a substantial impact on the subsequent subgroup symptom network analyses.

### 3.4 Symptom network analysis of the two latent classes of kinesiophobia in older adults with COPD

#### 3.4.1 Centrality analysis of symptom networks

Based on the partial correlation matrices of the 15 symptoms, symptom networks were constructed for the high-fear–somatic focus group (*n* = 368) and the lower-fear–activity avoidance group (*n* = 178), respectively (see Figures 2, 3). Three centrality indices, namely strength, closeness, and betweenness, were used to evaluate the relative importance of each symptom within the networks (see Figure 4). Because the stability of closeness and betweenness did not reach acceptable standards, strength was used as the primary criterion for identifying core symptoms, while closeness and betweenness were considered descriptive references only.

**Figure 2:**
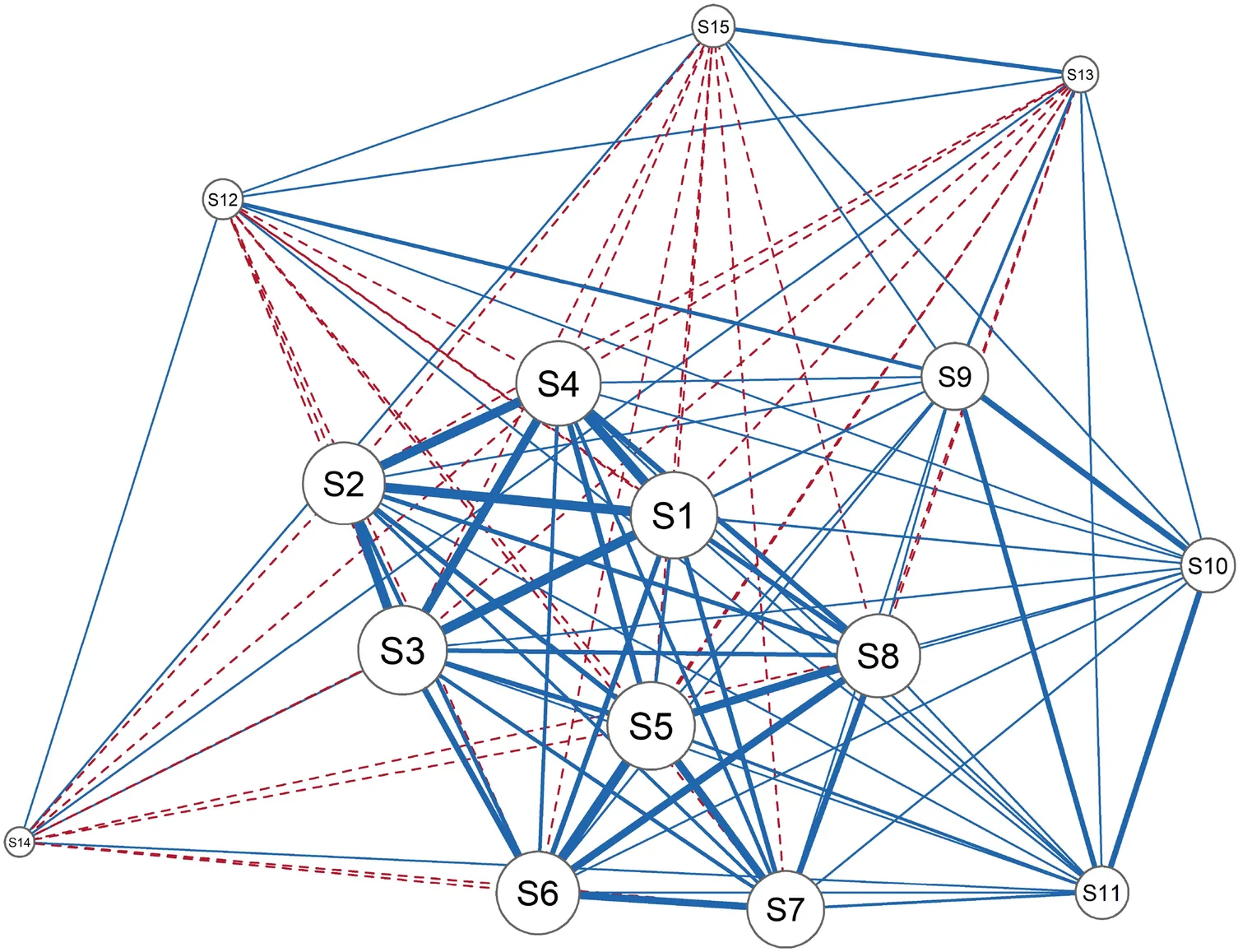
Symptom network of the high-fear-somatic focus group. S1 — Dyspnea; S2 — Fatigue; S3 — Cough; S4 — Sputum; S5 — Chest tightness; S6 — Leg weakness; S7 — Anxiety; S8 — Sleep disturbance; S9 — Appetite loss; S10 — Dry mouth; S11 — Social avoidance; S12 — Depression; S13 — Poor concentration; S14 — Constipation; S15 — Palpitations.

**Figure 3:**
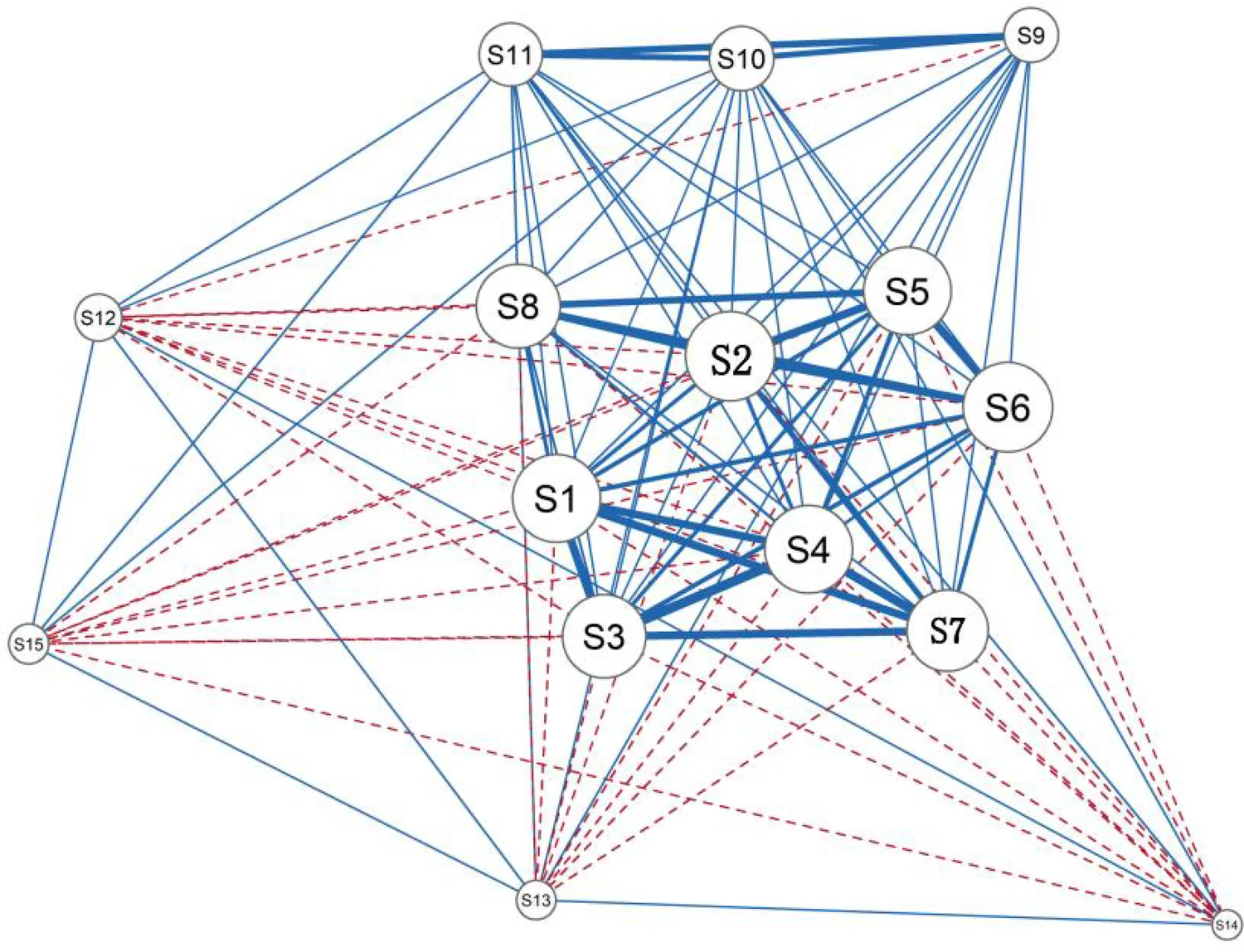
Symptom network of the lower-fear-activity avoidance group. S1 — Dyspnea; S2 — Fatigue ; S3 — Cough; S4 — Sputum; S5 — Chest tightness; S6 — Leg weakness; S7 — Anxiety; S8 — Sleep disturbance; S9 — Appetite loss; S10 — Dry mouth; S11 — Social avoidance; S12 — Depression; S13 — Poor concentration; S14 — Constipation; S15 — Palpitations.

**Figure 4:**
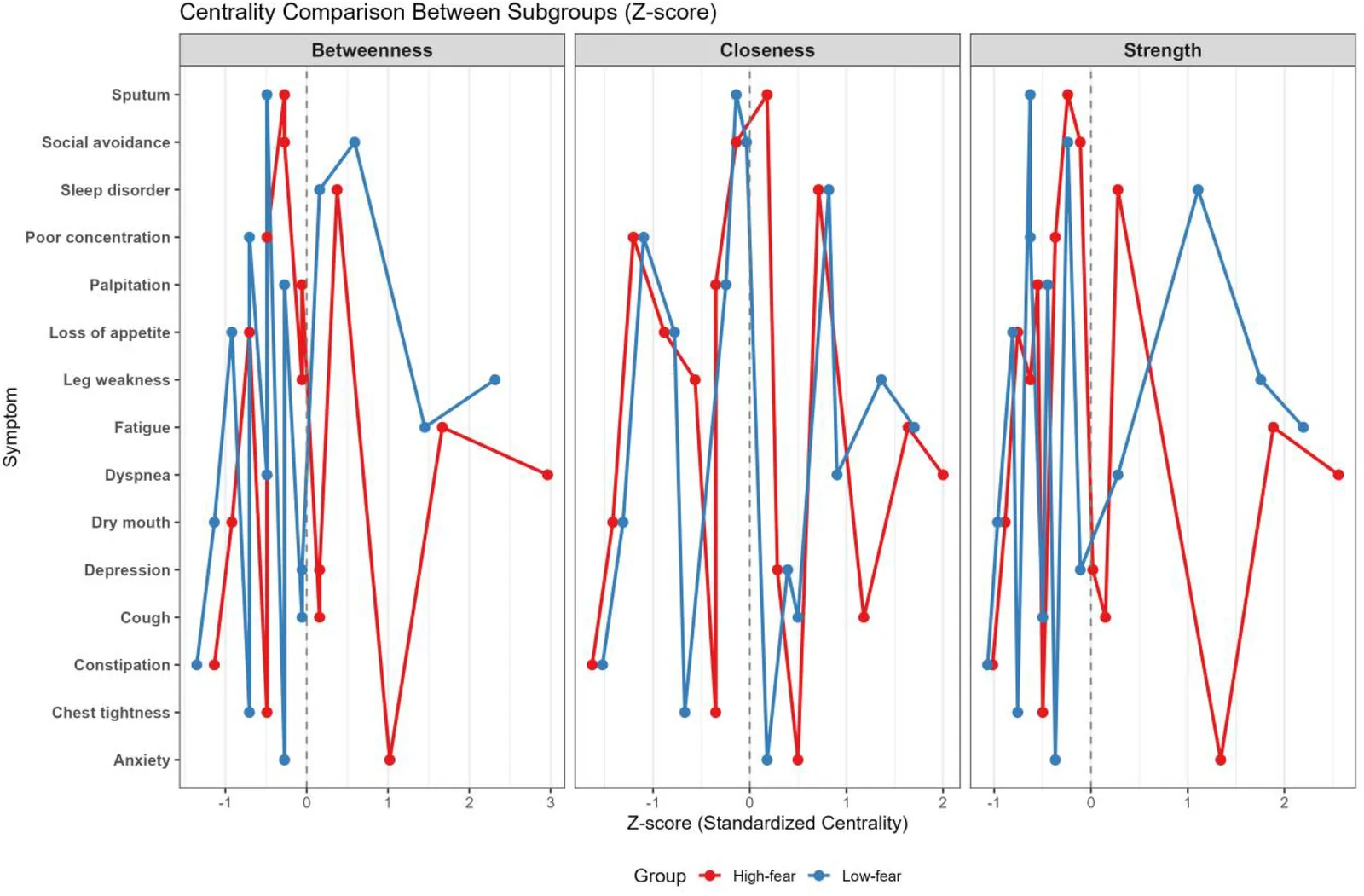
Centrality indices of the symptom networks for the high-fear-somatic focus group and the lower-fear-activity avoidance group.

In the high-fear–somatic focus group, the top three symptoms by strength were dyspnea (strength = 1.78), fatigue (strength = 1.52), and anxiety (strength = 1.31). The top three symptoms by closeness were dyspnea (closeness = 0.00621), fatigue (closeness = 0.00587), and cough (closeness = 0.00544). The top three symptoms by betweenness were dyspnea (betweenness = 21), fatigue (betweenness = 15), and anxiety (betweenness = 12). Based on the strength index, dyspnea ranked first and was identified as the core symptom of the symptom network in this group.

In the lower-fear–activity avoidance group, the top three symptoms by strength were fatigue (strength = 1.64), lower-limb weakness (strength = 1.47), and sleep disorder (strength = 1.22). The top three symptoms by closeness were fatigue (closeness = 0.00593), lower-limb weakness (closeness = 0.00561), and dyspnea (closeness = 0.00518). The top three symptoms by betweenness were lower-limb weakness (betweenness = 18), fatigue (betweenness = 14), and social avoidance (betweenness = 10). Based on the strength index, fatigue ranked first and was identified as the core symptom of the symptom network in this group.

#### 3.4.2 Stability of the symptom networks

The stability of the centrality indices was evaluated using the bootstrap method (1,000 iterations). In the high-fear-somatic focus group, the CS-coefficient for strength was 0.361 (>0.25, acceptable), the CS-coefficient for closeness was 0.218 (<0.25, unacceptable), and the CS-coefficient for betweenness was 0.052 (<0.25, unacceptable). In the low-fear-activity avoidance group, the CS-coefficient for strength was 0.334 (acceptable), the CS-coefficient for closeness was 0.192 (unacceptable), and the CS-coefficient for betweenness was 0.047 (unacceptable). These results indicate that the strength centrality index had acceptable stability in both networks, whereas the stability of closeness and betweenness was inadequate. Therefore, the strength index should be prioritized when interpreting core symptoms.

#### 3.4.3 Comparison of symptom networks between the two groups

The NetworkComparisonTest (NCT) was used to compare the symptom networks between the two groups. The results showed that the difference in network structure between the high-fear–somatic focus group and the lower-fear–activity avoidance group was not statistically significant (M = 0.31, *P* = 0.127), and the difference in global strength was also not statistically significant (S = 3.89, *P* = 0.094). These findings indicate that no statistically significant differences were detected in the overall network structure or global connectivity strength between the two groups in this study. However, it should be noted that *P* > 0.05 merely indicates a lack of evidence supporting the existence of group differences; it does not equal equivalence or similarity of the two network structures, nor can it be directly interpreted as evidence of shared symptom association patterns across the two subgroups. This null finding may be influenced by sample size, network estimation accuracy, and statistical power, and requires further verification in future studies with larger samples or equivalence testing.

## 4 Discussion

The present study found that the prevalence of kinesiophobia in older adult patients with COPD was 88.46%, which is relatively high and consistent with the range of 80%–90% reported in previous studies (20). This high prevalence suggests that kinesiophobia is an important psychological barrier to pulmonary rehabilitation participation in older adult patients with COPD and warrants clinical attention. Through latent profile analysis, this study classified kinesiophobia in older adult patients with COPD into two heterogeneous subgroups: a high-fear-somatic focus group (67.4%) and a lower-fear-activity avoidance group (32.6%). This classification goes beyond the traditional “presence/absence” dichotomy of kinesiophobia and reveals internal structural differences in kinesiophobia. Patients in the high-fear group exhibited particularly prominent catastrophic cognitions about dyspnea, believing that activity would aggravate their condition or cause physical harm. In contrast, patients in the lower-fear group had relatively lower fear levels but still showed a certain degree of avoidance behavior, possibly stemming from anticipatory concern about dyspnea after activity.

Symptom network analysis showed that dyspnea was the core symptom in the high-fear-somatic focus group, with its strength ranking first. This finding is consistent with the pathophysiological characteristics of COPD: due to airway obstruction and lung hyperinflation, patients are prone to experience dyspnea during daily activities, and this discomfort may be catastrophically interpreted by patients as “lung damage” or “disease worsening,” thereby reinforcing kinesiophobia (21). The fear-avoidance model posits that when patients equate dyspnea with a danger signal, they may develop maladaptive avoidance behaviors, forming a vicious cycle of “dyspnea → fear → avoidance → disability” (22). In the present study, patients in this group scored significantly higher on the somatic focus dimension than on the activity avoidance dimension, which, to some extent, supports the plausibility of this association pattern. Furthermore, anxiety also showed relatively high centrality in this group (strength ranked third and betweenness ranked third), suggesting a possible synergistic effect between anxiety and dyspnea: anxiety may enhance perceptual sensitivity to somatic symptoms, making patients more likely to experience mild breathlessness as a threat, thereby exacerbating kinesiophobia. This is consistent with previous findings that anxiety is positively correlated with kinesiophobia in patients with COPD (23). It should be emphasized that the above pathways are speculative explanations based on cross-sectional correlation networks, and their causal directions need to be verified by longitudinal studies.

The core symptom of the lower-fear–activity avoidance group was fatigue, with its strength ranking first. This finding suggests that kinesiophobia in this group may not originate from a strong fear of dyspnea, but rather from activity avoidance driven by persistent fatigue. Fatigue in patients with COPD is multifactorial (24, 25): on the one hand, chronic hypoxia and increased work of breathing lead to excessive energy expenditure; on the other hand, systemic inflammation, malnutrition, and sleep disturbance jointly aggravate the sense of fatigue. Fatigue deprives patients of the motivation and confidence to be physically active, and even when their fear level is not high, they may choose to avoid activity simply because they “have no energy.” Notably, lower-limb weakness ranked first in betweenness in this group and showed a strong association with fatigue. However, because the stability of betweenness was insufficient and cross-sectional network analysis cannot distinguish temporal relationships among symptoms, lower-limb weakness should not be regarded as a “bridge symptom” connecting other symptoms. The association between lower-limb weakness and fatigue may reflect a bidirectional influence: fatigue may lead to reduced activity and consequently lower-limb weakness, or lower-limb weakness may limit activity and thereby aggravate fatigue (26). Sleep disorder ranked third in strength in this group, which may be related to nocturnal dyspnea, cough, or anxiety, and may further aggravate daytime fatigue (27). Although the core symptoms differed between the two groups, the network comparison test showed that the differences in network structure and global connectivity strength were not statistically significant (*P* > 0.05). This result suggests that there may be common connectivity patterns across subgroups in the symptom networks of older adult patients with COPD. For example, strong associations between dyspnea and fatigue, and between fatigue and anxiety, may be universal regardless of kinesiophobia level. Nevertheless, the differences in core symptoms imply that different subgroups may require different intervention emphases, although this hypothesis needs to be further verified by interventional studies.

Based on the above cross-sectional findings, it should be emphasized that the core symptoms identified by latent profile analysis and symptom network analysis only represent the central symptoms within each subgroup's symptom network. They may serve as potential reference indicators worthy of attention in future intervention research, but should not be regarded as the sole determinants of individualized protocols, nor should they be inferred as the causes of nonadherence to pulmonary rehabilitation. The following intervention strategies constitute a preliminary candidate framework developed on the basis of GOLD and ATS/ERS pulmonary rehabilitation guideline principles, combined with evidence from cognitive-behavioral interventions for dyspnea-related kinesiophobia in COPD, and informed by the symptom network characteristics of each subgroup identified in this study. This framework is proposed for reference in the design of future randomized controlled trials. For the high-fear-somatic focus group, network analysis showed that dyspnea had the highest strength centrality, and scores on the somatic focus dimension were prominent, suggesting that dyspnea-related catastrophic cognition may be a candidate target deserving priority in intervention design for this group; however, its exact causal role still needs to be verified by longitudinal studies. Therefore, candidate intervention directions focusing on dyspnea-related cognitive management and graded exercise exposure may be considered (28, 31): ① Cognitive restructuring: through health education, help patients understand that exercise-induced dyspnea is a normal physiological response rather than a sign of lung injury, thereby breaking the catastrophic thinking pattern; ② Graded exercise exposure: starting with low-intensity activities such as in-bed limb exercises and bedside standing, gradually progressing to walking, cycling, and other aerobic exercises, allowing patients to experience in a safe environment that “dyspnea can be alleviated after exercise,” thereby building exercise confidence; ③ Breathing training: pursed-lip breathing and diaphragmatic breathing training can improve respiratory efficiency and reduce exertional breathlessness, thereby lowering fear levels; ④ Relaxation training: addressing anxiety symptoms through progressive muscle relaxation or meditation practices to reduce sympathetic nervous system excitability.

For the lower-fear–activity avoidance group, network analysis showed that fatigue had the highest strength centrality, and there were strong associations among fatigue, lower-limb weakness, and activity avoidance. This association suggests that fatigue and lower-limb functional insufficiency may be potential directions worthy of attention in future intervention design for this group, but it does not imply that fatigue directly causes avoidance or disability. Therefore, candidate intervention directions focusing on fatigue management and lower-limb functional exercise may be considered (32, 35): ① Energy conservation techniques: guide patients to reasonably plan their activity rhythm, break tasks into smaller components, and take intermittent rest to reduce unnecessary energy expenditure; ② Nutritional support: conduct nutritional assessment for patients at risk of malnutrition, and supplement protein and vitamins when necessary to support energy metabolism and muscle function; ③ Lower-limb muscle strength training: given the network association between lower-limb weakness and fatigue, explore the effects of resistance training, such as seated knee extension, straight-leg raises, and marching in place, on improving lower-limb function and exercise tolerance; ④ Sleep hygiene education: recommend regular sleep schedules, avoidance of caffeine intake before bedtime, and optimization of the bedroom environment, with referral to sleep specialists when necessary; ⑤ Social support: encourage patients to participate in pulmonary rehabilitation groups or online communities to reduce social withdrawal through peer support. It should be re-emphasized that the above intervention framework is neither a direct excerpt from existing guidelines nor a validated clinical protocol, but rather a preliminary research hypothesis developed by integrating guideline principles, existing intervention evidence, and the symptom network characteristics identified in this study. Its feasibility, safety, and efficacy need to be verified through rigorous randomized controlled trials.

In addition, Table 1 showed that only 24.5% of the older adult patients with COPD in this study had ever received pulmonary rehabilitation guidance, meaning that 75.5% had never received systematic rehabilitation education. This high proportion of “lack of guidance” warrants close attention. For patients without adequate pulmonary rehabilitation education, they often do not understand the nature of exertional dyspnea or fatigue, and are more likely to catastrophically interpret post-activity breathlessness or tiredness as signs of “lung damage” or “disease worsening,” thereby reinforcing kinesiophobia. In the absence of appropriate cognitive guidance, patients are also more likely to adopt a conservative strategy of avoiding activity to prevent discomfort, which parallels the characteristics of the two subgroups identified in this study—one dominated by dyspnea catastrophizing and the other by fatigue-driven avoidance. Although the cross-sectional design precludes causal inference, these data suggest that the high prevalence of kinesiophobia may be related not only to the disease itself but also to the systematic lack of pulmonary rehabilitation education. Therefore, in clinical practice, in addition to early screening for kinesiophobia, greater efforts should be made to provide pulmonary rehabilitation-related health education, particularly covering dyspnea management, explanations of the safety of physical activity, and the benefits of exercise.

The present study has several limitations. First, the cross-sectional design cannot establish causal relationships, and the dynamic evolution between kinesiophobia and symptoms needs to be further clarified in longitudinal studies. Second, the sample was derived from a single center, which may introduce selection bias, and the generalizability of the conclusions needs to be verified through multicenter studies. Third, symptom data were all based on patient self-reports, which cannot completely avoid recall bias. Fourth, the stability of network centrality indices varied: bootstrap tests showed that strength centrality reached acceptable standards, but the stability of closeness and betweenness was unsatisfactory. Therefore, strength was used as the primary criterion for identifying core symptoms, while closeness and betweenness were considered descriptive references only. In addition, although the overall sample size met the basic requirements for network analysis, the sample size of the lower-fear–activity avoidance group (*n* = 178) was relatively small and did not reach the recommended standard of 5 cases per parameter, which may have affected the stability and precision of network estimation in this group.

It should be noted that disease severity and smoking status may also influence the central role of dyspnea in the network. In this study, patients with GOLD grade Ⅲ–Ⅳ accounted for 50.4%, and current smokers accounted for 46.9%. These factors might theoretically enhance the prominence of dyspnea symptoms. However, stratified network analyses by GOLD grade or smoking status were not performed in this study, primarily because latent profile analysis had already yielded two subgroups, and further stratification would have substantially reduced the sample size of each subgroup, making it difficult to ensure the stability and precision of network estimation. Nevertheless, the strength centrality of dyspnea in the high-fear group (1.78) was considerably higher than that of the second-ranked symptom (1.52), suggesting that its central role is unlikely to be entirely explained by disease severity or smoking status. In addition, this study adopted a two-step analytical strategy, in which participants were first assigned to a single class using the maximum posterior probability method, after which symptom networks were estimated independently within each subgroup, without explicitly incorporating posterior classification probabilities into the network estimation. Although the average posterior probabilities and entropy values indicated high classification quality, this strategy may still underestimate the influence of classification error on the network results. Future studies may consider robust three-step approaches (e.g., the BCH method) or probability-weighted network analyses to more rigorously evaluate the impact of classification uncertainty.

Regarding hospitalization status, all patients included in this study completed the survey during clinical stability; hospitalized patients were assessed after their condition had stabilized prior to discharge, rather than being enrolled during an acute exacerbation. Future studies should further expand the sample size and construct and compare networks separately for patients with GOLD grade Ⅰ–Ⅱ versus Ⅲ–Ⅳ, and for smokers versus non-smokers, in order to clarify the potential moderating effects of the above factors and to verify the stability of the symptom network structure in the lower-fear group.

## 5 Conclusion

This study used latent profile analysis to classify kinesiophobia in older adult patients with COPD into two heterogeneous subgroups. Symptom network analysis further revealed differences in core symptoms between the two groups: in the high-fear group, dyspnea was the core symptom, ranking first in strength, closeness, and betweenness; in the lower-fear group, fatigue was the core symptom, ranking first in strength and closeness, and second in betweenness. Based on these findings, the study proposed targeted pulmonary rehabilitation intervention strategies: for the high-fear group, interventions should focus on dyspnea management and cognitive-behavioral intervention, helping patients rebuild correct cognition about dyspnea and break the fear-avoidance cycle through graded exercise exposure; for the lower-fear group, interventions should focus on fatigue management and lower-limb muscle strength training, supplemented by energy conservation techniques and sleep hygiene education. This study provides preliminary theoretical and practical references for subtype identification and individualized intervention of kinesiophobia in older adult patients with COPD. Future longitudinal studies are needed to further clarify the causal relationships between core symptoms and kinesiophobia, and to develop and validate subtype-based personalized pulmonary rehabilitation programs.

## Data Availability

The original contributions presented in the study are included in the article/Supplementary Material, further inquiries can be directed to the corresponding authors.
